# The association between crowding within households and behavioural problems in children: Longitudinal data from the Southampton Women’s Survey

**DOI:** 10.1111/ppe.12550

**Published:** 2019-04-29

**Authors:** Rachael Marsh, Theodosia Salika, Sarah Crozier, Sian Robinson, Cyrus Cooper, Keith Godfrey, Hazel Inskip, Janis Baird, for the SWS Study Group

**Affiliations:** ^1^ Medical Research Council Lifecourse Epidemiology Unit, Southampton General Hospital University of Southampton Southampton UK; ^2^ NIHR Southampton Biomedical Research Centre, University Hospital Southampton NHS Foundation Trust University of Southampton Southampton UK

**Keywords:** behaviour, cohort study, crowding, housing tenure, parent‐child interactions, strengths and difficulties score

## Abstract

**Background:**

In England, nearly one child in ten lives in overcrowded housing. Crowding is likely to worsen with increasing population size, urbanisation, and the ongoing concerns about housing shortages. Children with behavioural difficulties are at increased risk of mental and physical health problems and poorer employment prospects.

**Objective:**

To test the association between the level of crowding in the home and behavioural problems in children, and to explore what factors might explain the relationship.

**Methods:**

Mothers of 2576 children from the Southampton Women's Survey population‐based mother‐offspring cohort were interviewed. Crowding was measured at age 2 years by people per room (PPR) and behavioural problems assessed at age 3 years with the Strengths and Difficulties Questionnaire (SDQ). Both were analysed as continuous measures, and multivariable linear regression models were fitted, adjusting for confounding factors: gender, age, single‐parent family, maternal education, receipt of benefits, and social class. Potential mediators were assessed with formal mediation analysis.

**Results:**

The characteristics of the sample were broadly representative of the population in England. Median (IQR) SDQ score was 9 (6‐12) and PPR was 0.75 (0.6‐1). In households that were more crowded, children tended to have more behavioural problems (by 0.20 SDQ points (95% CI 0.08, 0.32) per additional 0.2 PPR, adjusting for confounding factors). This relationship was partially mediated by greater maternal stress, less sleep, and strained parent‐child interactions.

**Conclusions:**

Living in a more crowded home was associated with a greater risk of behavioural problems, independent of confounding factors. The findings suggest that improved housing might reduce childhood behavioural problems and that families living in crowded circumstances might benefit from greater support.

**Synopsis**

**Study question**

Is there an association between the level of crowding in the home and behavioural problems in children, and if so, what factors might explain the relationship?

**What’s already known**

Early, small scale studies indicate that living in a more crowded home is associated with a greater risk of behavioural problems in children.

**What this study adds**

This UK‐based cohort study confirms that living in a more crowded home is associated with a greater risk of behavioural problems in children, independent of confounding factors (gender, age, single‐parent family, maternal education, receipt of benefits and social class and neighbourhood quality). The relationship was mediated in‐part by maternal stress, less sleep, and strained parent‐child interactions. Crowding occurs more commonly in social housing.

## 1 INTRODUCTION

Behavioural problems lead to a range of negative outcomes including mental and physical health problems,^1^ increased violence and risk of a criminal conviction,^2^ and poorer educational attainment and employment prospects.^1^ Studies have shown that behavioural problems affect one in ten children in the United Kingdom (UK).^1^, ^3^ This results in a serious burden for the individual, their families, and the wider community and economy.

Housing quality is now widely recognised as one of the social determinants of health.^4^ Determining which elements of housing quality can be detrimental to behavioural problems in children could enable policies to be more effectively targeted at addressing this inequity. One such important and timely element is crowding. Crowding is worsening in the current housing crisis,^5^ and new homes in the UK are the smallest in Western Europe.^6^

**Figure 1:**
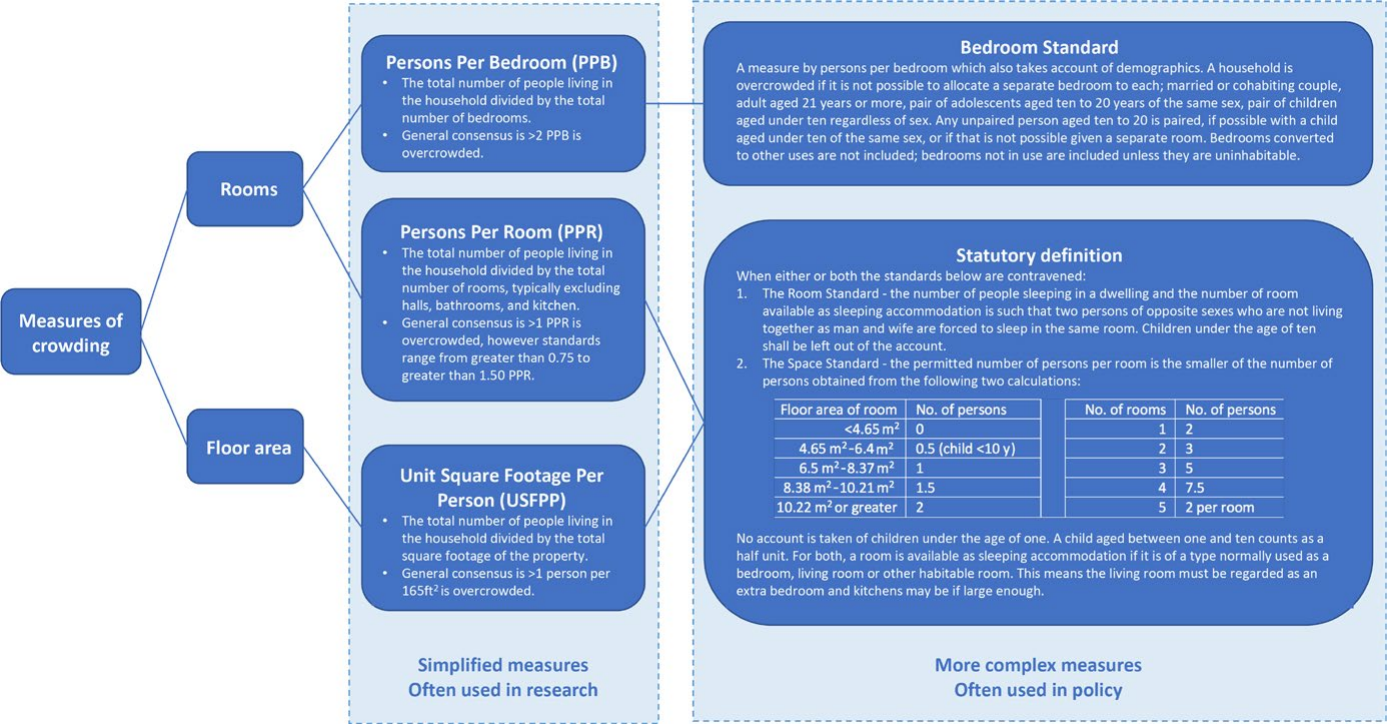
Summary of measures of crowding and definitions of overcrowding, the association between crowding within households and behavioural problems in children, Southampton, 2019^7^, ^35^

There are various ways both to measure the level of crowding in a household and to define the point at which a household is classed as overcrowded (see Figure 1 for definitions). People per room (PPR) is the most useful measure of crowding as it is continuous and is the most commonly used metric in research.^7^ The bedroom standard is widely used as a definition for classifying a household as overcrowded.^8^ Using the bedroom standard, nearly one million children, or one child in every ten, live in overcrowded conditions in England.^8^, ^9^, ^10^ This problem is more common among families of lower socio‐economic status, in rented accommodation, and in cities, with nearly one child in every three living in an overcrowded home in London's social housing.^5^, ^10^

Most research on the effects of crowding is based on adults.^11^ Yet children are particularly influenced by their home environment.^12^ Studies have shown crowding in the home has a negative impact on children's education and a range of physical health outcomes,^13^ but, as highlighted by other researchers, despite the strong theoretical links to adverse psychological processes, almost no research on children has focused on associations between crowding and behavioural outcomes.^14^

The majority of studies on crowding in the home and behavioural problems in children originate from America, are from the 1970s or earlier, were based on very small samples, and used cross‐sectional designs.^13^, ^14^, ^15^, ^16^ Notably, there has not been a study in the UK for over 25 years.^14^, ^15^ In most of the studies, children living in crowded households had more behavioural problems than children in less crowded households.^14^, ^15^, ^16^, ^17^, ^18^ Crowding may impact on children's behaviour through a lack of privacy or space to play,^19^, ^20^ increased reliance on childcare,^1^ interrupted sleep,^17^ or impacts on parent‐child interactions including conflict, reduced monitoring, and less parental responsiveness.^1^, ^16^, ^21^ Despite the numerous theoretical explanations for the relationship between crowding and child behaviour, very little research has included potential confounding or mediating factors.

The aim of this study was to assess whether the level of crowding in the home is associated with more behavioural problems in a UK cohort of children, and to explore what factors might explain the relationship.

## 2 METHODS

### 2.1 Participants

**Figure 2:**
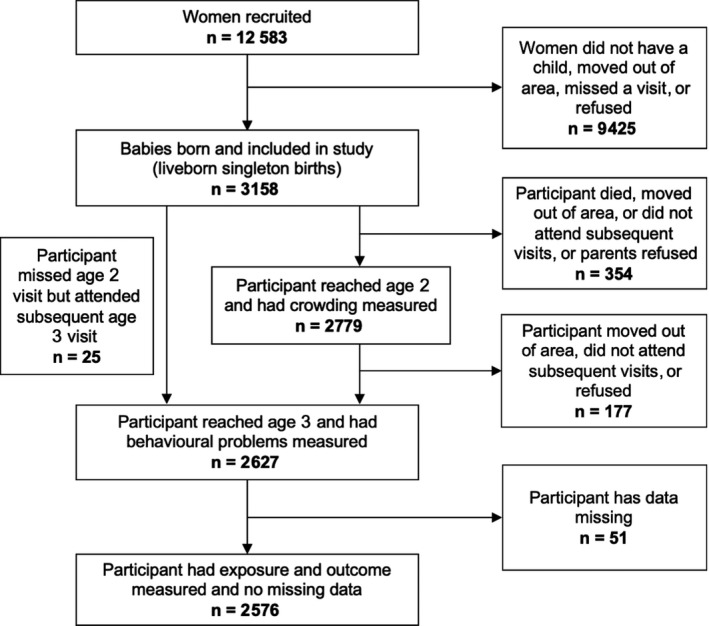
Participant flow diagram and dropout at various stages of the Southampton Women's Survey, the association between crowding within households and behavioural problems in children, Southampton, 2019

The Southampton Women's Survey (SWS) is a prospective cohort study of 12 583 women aged 20‐34 years recruited, when not pregnant, from the general population resident in Southampton.^22^ A total of 3,158 women who subsequently became pregnant were followed through their pregnancy, and their children were then followed up at intervals during childhood. Those who had information collected on behavioural problems at age 3 years were included in the study. The final sample consisted of 2576 children (see Figure 2). Information relating to the children in this study was collected from 2001 to 2010. The study had full approval from the Southampton and Southwest Hampshire Local Research Ethics Committee, and all participants’ mothers gave written informed consent.

The level of crowding in the household at age 2 years was captured as PPR. Information on the numerator (sum of the number of people living in the household) and the denominator (total number of rooms, excluding halls and bathrooms, minus one to represent the kitchen) was collected during face‐to‐face interviews with the participants’ mothers at their homes. A further question assessed whether the household composition had changed since pregnancy. Behavioural problems were assessed at age 3 years using the preschool, parent‐only version of the Strengths and Difficulties Questionnaire (SDQ). Mothers were questioned regarding their children in four areas: emotional, conduct, hyperactivity/inattention, and peer problems; and the scores from each of these were summed to create a total difficulties score.^23^ This score can range from 0 to 40 and was treated as a continuous variable. A higher score indicates greater behavioural problems (a score under 13 is “close to average,” 13‐15 “slightly raised,” 16‐18 “high,” and 19 and above “very high”).^24^

**Figure 3:**
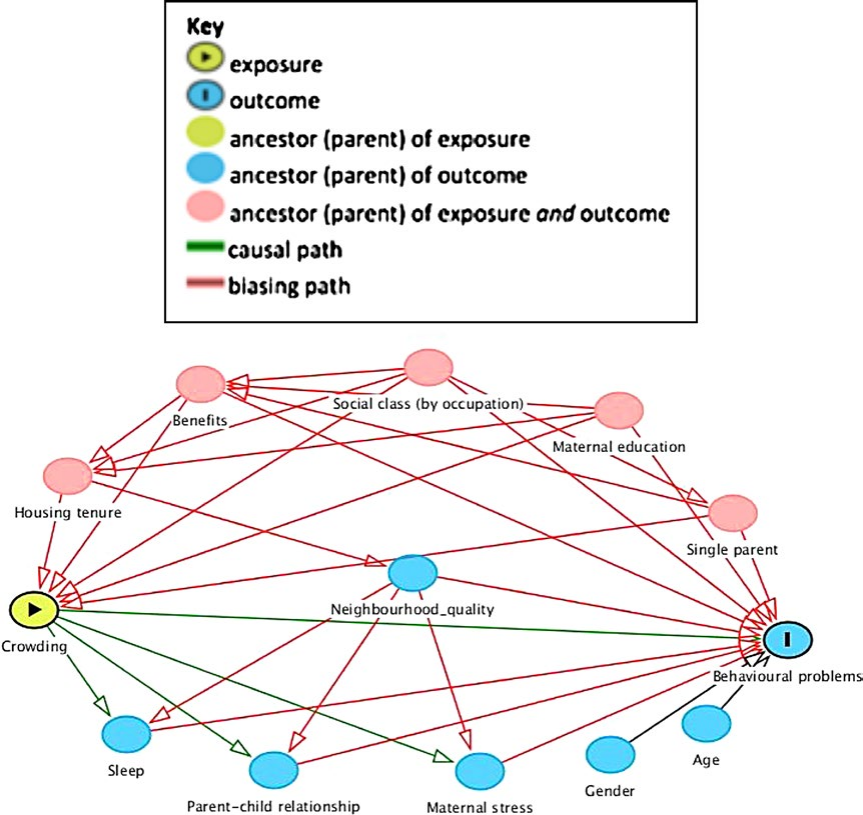
DAG model created to show covariates included in the analyses, the association between crowding within households and behavioural problems in children, Southampton, 2019

Potential confounding factors were identified a priori from existing literature and included in a directed acyclic graph (DAG) (see Figure 3). This indicated two different minimal sufficient adjustment sets. The first included level of maternal educational attainment, highest level of parental social class (by occupation), single‐parent household, whether the household received benefits (support/job seekers allowance, working tax credit, or housing benefits), and housing tenure. The second included the same factors with the exception of housing tenure which was replaced with neighbourhood quality. Additionally, adjustments for age and gender of the child were included in all analyses to improve the precision of the outcome variable. We separately examined the relationship between housing tenure and crowding to try to identify the types of housing in which most crowding occurs. Housing tenure was classified as owner occupied (homes owned outright and mortgaged); privately rented; socially rented (housing rented from local authorities and housing associations); or other (families who live with a relative, in a hostel, halls of residence, or bed and breakfast).

The following variables, shown in the DAG, were considered as possible mediators: sleep duration (time spent asleep per night); maternal stress (stress experienced in daily living in the last 4 weeks ranked on a 5‐point scale); and two variables for parent‐child interactions (conflict and closeness) which were measured using the Child‐Parent Relationship Scale (CPRS). CPRS is a self‐report instrument, completed by mothers, that assesses their perceptions of their relationship with their child. It is widely used and has been validated for use at this age.^25^ It produces conflict and closeness scores which run from 0 to 60, with higher scores representing negative and positive interactions, respectively.

Information on all the confounding and mediating variables and housing tenure was collected in the same interview with the mothers of the participants when the children were aged 2 years, with the exception of parent‐child interactions and sleep, which were measured in the interview at age 3 years.

### 2.2 Statistical analysis

Using Stata 15.0,^26^ standard summary statistics including median, interquartile range (IQR), or number (n) and percentage were produced for the variables in the analysis. Spearman's correlation and linear regression methods were used to explore the relationship between crowding and behavioural problems. In all the models, crowding was entered in units of 0.2 PPR which equates to an additional person in an average‐sized five‐room household. The first model simply adjusted for child's gender and age. Models 2 and 3 were based on the two options for minimal sufficient adjustment indicated by the DAG. In Model 2, single parent, maternal education, receipt of benefits, social class, and housing tenure were included. In Model 3, neighbourhood quality replaced housing tenure while the other variables remained the same.

Mediation analysis, using formal mediation techniques, for the association between crowding and SDQ score was implemented.^27^, ^28^ We used Model 3 to consider the mediators. Bias‐corrected confidence intervals were estimated from 500 Monte Carlo draws for nonparametric bootstrap. Direct and indirect effects were averaged across all individuals.

Data on behavioural problems were slightly skewed to the right so a sensitivity analysis was conducted using the square‐root transformation. We tested for nonlinearity of the relationship between child's behaviour and crowding by including a quadratic term for crowding in our models. Further, we conducted an analysis restricted only to those living in owner‐occupied houses.

In our data set, 78% of individuals had fully observed data. The proportion of missing data for each variable ranged from 0.2% (gender) to 19% (conflict score); we did not identify important missing data patterns in our data set. We used multiple imputation of missing data to minimise selection bias and increase the power of our analysis. For each imputation model, we included all the variables identified from the DAG as potential confounders or mediators, as well as our outcome. We generated 100 imputed data sets and combined the coefficient estimates using Rubin's rule.^29^, ^30^ We based our imputations on the assumption that missingness in the data is explained by the observed variables included in the imputation model (ie data are missing at random).^31^ More details are in Table S1.

## 3 RESULTS

The characteristics of the 2576 children are given in Table 1. The median age was 3 years at the time of assessment of behavioural problems. The study sample characteristics were almost identical to the wider SWS cohort and broadly in line with England figures.^1^, ^5^, ^23^

**Table 1:** Baseline characteristics of the study population, the association between crowding within households and behavioural problems in children, Southampton, 2019

Participant characteristics	Study sample n = 2576 Median (IQR) or *n (%)*
Crowding (PPR)	0.75 (0.60, 1.00)
Behavioural problems (SDQ score)	9 (6, 12)
Boys	1338 (52)
Age (years)	3.04 (3.01, 3.09)
Single‐parent household	231 (9)
Maternal White ethnicity	2478 (96)
Maternal education^a^	
No qualifications	66 (3)
GCSE only	939 (37)
A‐levels or equivalent	825 (32)
Degree or higher	740 (29)
In receipt of benefits	871 (34)
Housing tenure^b^	
Owner occupier	2046 (79)
Privately rented	125 (5)
Socially rented	326 (13)
Other	78 (3)
Social class	
Professional (I)	303 (12)
Management and technical (II)	1258 (49)
Skilled nonmanual (IIIN)	662 (26)
Skilled manual (IIIM)	240 (9)
Partly skilled (IV)	96 (4)
Unskilled (V)	14 (1)
Parent‐child interaction^c^	
Conflict	25 (20, 30)
Closeness	45 (43, 47)
Sleep duration (hours per night)	11.0 (10.5, 11.5)
Mothers level of stress^d^	
None	331 (13)
Mild	1715 (66)
Moderate to severe	525 (20)

Percentage totals may not add to 100 due to rounding. Only data on behavioural problems were slightly skewed, but medians (IQRs) are presented for consistency.

a ISCED level equivalents are as follows: No qualifications is ISCED‐0, 1, and 2; GCSE only is ISCED‐3 A‐levels or equivalent ISCED‐3 and 4; and Degree or diploma is ISCED‐4, 5, and 6.

b Owner occupied (homes owned outright and mortgaged), socially rented (housing rented from local authorities and housing associations), and other (family lives with a relative, in a hostel, halls of residence, or bed and breakfast).

c Child‐Parent Relationship Scale produces conflict and closeness scores which run from 0 to 60, with higher scores representing negative and positive interactions between parent and child, respectively.

d Mothers ranked the stress or pressure they experience in daily living in a 4‐week period on a 5‐point scale: none, just a little, a good bit, quite a lot, or a great deal. Responses were grouped so that “just a little” and “a good bit” represent mild stress and “quite a lot” and “a great deal” represent moderate‐to‐severe stress.

In households, the number of rooms ranged from 2 to 12 with a mean of 6.0. The number of individuals in households ranged from 2 to 11, and level of crowding ranged from 0.3 to 4 PPR. There was relatively little change in the level of crowding from the child's birth to age 2 years, with 1951 (76%) households having no change to the number of individuals in them. Of households that did see a change, the majority were due to the addition of a single child. The total difficulties behavioural score ranged from 0 to 31, with 248 (9.6%) of children having “high” or “very high” scores (SDQ score ≥ 16).

Table 2, Model 1 shows the positive association between crowding and behavioural problems adjusted for age and gender. In Model 2, which also includes additional adjustment for the confounding variables (single‐parent households, maternal education, income, social class, and housing tenure), the association between behavioural problems and crowding was markedly attenuated. In Model 3, in which housing tenure was replaced by neighbourhood quality, there was less attenuation from Model 1 than was seen in Model 2. In households that were more crowded by 0.2 PPR (equating to an additional person in an average‐sized five‐room household), the children tended to have more behavioural problems by 0.20 SDQ points (95% CI 0.08, 0.32, *P* < 0.001), after adjustment for confounding factors. Furthermore, children with SDQ scores ≥ 16 (“high” or “very high” total difficulties score) lived in houses that had, on average, 0.2 more PPR than children with SDQ scores < 13 (“close to average” score). Examining the subscales of the SDQ score indicated that the association was dominated by the relationship with conduct problems and peer problems rather than with the other subscales of hyperactivity and emotional symptoms (Table S2).

**Table 2:** Multivariable regression assessing the relationship between crowding in the household and behavioural problems in children, the association between crowding within households and behavioural problems in children in the multiply imputed data set, Southampton, 2019

Variable	Model 1 (n = 2,576)	Model 2 (n = 2,566)	Model 3 (n = 2,563)
	β (95% CI)	β (95% CI)	β (95% CI)
Crowding (0.2 PPR)	0.45 (0.34, 0.56)	0.13 (−0.003, 0.26)	0.20 (0.08, 0.32)
Girls (vs boys)	−1.03 (−1.37, −0.68)	−1.06 (−1.40, −0.72)	−1.04 (−1.38, −0.70)
Childs age (years)	−0.70 (−2.64, 1.19)	−1.53 (−3.44, 0.37)	−1.49 (−3.38, 0.41)
Single parent		−0.33 (−0.99, 0.33)	−0.69 (−1.32, −0.07)
Maternal education		−0.33 (−0.47, −0.18)	−0.36 (−0.50, −0.21)
On benefits		0.28 (−0.11, 0.68)	0.32 (−0.07, 0.72)
Social class (by occupation)^a^		0.24 (0.04, 0.44)	0.26 (0.07, 0.46)
Housing tenure			
Owner occupier		0.00 (Reference)	
Privately rented		0.11 (−0.73, 0.94)	
Socially rented		1.54 (0.88, 2.19)	
Other		1.73 (0.71, 2.74)	
Neighbourhood quality^b^			0.21 (0.14, 0.28)
Constant	11.12	15.68	15.43

Model 1 is adjusted for child's gender and age Model 2 is adjusted for confounders in model 1 plus additional DAG‐identified confounders including single parent, maternal education, receipt of benefits, social class, and housing tenure Model 3 is adjusted for confounders in model 2, plus neighbourhood quality but excludes housing tenure.

a Ordered categorical variables included in the model as continuous variables to account for the trend.

b Summed ratings for eight categories: vandalism, litter, small, muggings, burglaries, disturbances, traffic, and noise. Possible score ran from 0 to 16 with a higher score indicating more problems.

The analysis of the multiply imputed data sets to take account of missing data found very similar results to those in Table 2. The results are given in Table S3.

The four mediators examined (conflict and closeness in the parent‐child relationship, maternal stress, and child sleep duration per night) explained 15% of the effect of crowding on behaviour. In the fully adjusted model, including all variables in Model 3 and all of the mediators, the coefficient for crowding (using the 0.2 PPR values) reduced to 0.16 (95% CI 0.04, 0.28) (see Table 3). This indicates that all of these factors could, in part, explain the positive association between crowding and behavioural problems, but that after adjustment, the relationship between crowding and behavioural problems remained.

**Table 3:** Regression analyses of potential mediators and associated factors in the relationship between crowding in the household and behavioural problems in children, the association between crowding within households and behavioural problems in children, Southampton, 2019

Covariate	Coefficient for crowding adjusted for confounders as in Model 3, further adjusted for each mediator	Coefficient for crowding adjusted for confounders as in Model 3, further adjusted for all mediators
Increasing stress^a^	0.19 (95% CI 0.06, 0.32)	0.16 (95% CI 0.04, 0.28)
Reduced sleep duration^b^	0.19 (95% CI 0.05, 0.33)	
Parent‐child interaction^c^		
Increasing conflict	0.19 (95% CI 0.07, 0.31)	
Increasing closeness	0.16 (95% CI 0.04, 0.28)	

Numbers rounded to two decimal places.

a Mothers ranked the stress or pressure they experience in daily living in a 4‐week period on a 5‐point scale: none, just a little, a good bit, quite a lot, or a great deal.

b Hours spent asleep per night.

c Child‐Parent Relationship Scale produces conflict and closeness scores which run from 0 to 60, with higher scores representing negative and positive interactions between parent and child, respectively.

A sensitivity analysis using a square‐root transformation of the data on behavioural problems produced the same Spearman's correlation coefficient and significance for the correlation between crowding and behavioural problems. All the same factors remained statistically significant in the regression analyses in Models 1 and 2. We found no evidence of nonlinearity in the relationships.

The association between crowding and housing tenure was found to be strong, with children living in socially rented housing being more likely to experience crowding (see eFigure 1). Some 25% of the variability in crowding was explained by housing tenure. Restricting the analysis to those living in owner‐occupied homes showed that even in such homes, there was an association between crowding and child's behaviour with the coefficient for crowding being 0.15 (95% CI −0.0006, 0.30).

## 4 COMMENT

### 4.1 Principal findings

This UK‐based study confirms the associations shown in studies in other countries that children living in crowded households had more behavioural problems than children in less crowded households and this was independent of age, gender, single‐parent households, and maternal education, receipt of benefits, and social class. It adds to the evidence base by showing that maternal stress, less sleep per night, and strained parent‐child interactions might all, in part, be mediating factors. Furthermore, we identified that children living in social housing tended to live in more crowded homes, but that even in owner‐occupied homes, crowding and behavioural problems are associated.

The findings of this study are consistent with the majority of earlier, small‐scale studies on crowding and behavioural problems and offer resolution to a number of common limitations, not least study design.^14^, ^15^, ^16^, ^17^, ^18^ It has a large sample size, strong, prospective cohort design, and relatively robust control for potential confounding factors. The findings agree with the only other longitudinal study to date by Solari et al,^12^ which also found that children from more crowded households had more behavioural problems than children from less crowded households, irrespective of socio‐economic status and demographic factors.

### 4.2 Strengths of the study

Possible reasons why the findings of this study differ from the few studies that did not find an association between crowding and behaviour, such as Li et al,^20^ are because of the differing methods of measuring crowding. Li et al used unit square footage per person; however, capturing crowding through PPR is preferred because it is has been reported as the most consistent crowding metric with human consequences,^7^ and because of inconsistencies in how people define bedrooms.^12^, ^16^ There is no known threshold for any detrimental effect from crowding on a child's behaviour, so the continuous measure is justified and more sensitive than arbitrary categorical intervals.^12^

A further strength of this study was its prospective cohort design. The longitudinal nature of the data enabled account to be taken of temporality. The SWS cohort has been well characterised, thus allowing consideration of important confounding factors, albeit that there is likely to be residual confounding. The characteristics of the sample were almost identical to the wider SWS cohort, but the SWS cohort is slightly more affluent than the general population in the UK, as commonly results from selection bias in studies.^23^ Interviewers and participants were blinded to the research hypothesis, which minimised reporting bias. Missing data did not seem to be a major problem as analyses of our multiply imputed data sets gave very similar results to the complete‐case analysis. The SDQ is not a clinical assessment, but it is a validated tool to measure behavioural problems in the sample age group.^32^ The age of 3 years was an appropriate time to measure the outcome as child behaviour shows increasing stability from around this point onwards.^1^

### 4.3 Limitations of the data

Several covariates could have been more refined; for example, receipt of benefits is a crude measure of income, and there is some evidence to suggest that the SDQ might be a more sensitive measure of behavioural problems after age 4 years.^32^ The exposure, outcome, and covariates were all reported by the participants’ mothers, which introduces the potential for response bias. For example, if some mothers in overcrowded households gave information that led to an underestimation of the PPR, then this might have led to an exaggerated effect size. However, the interviews were conducted in the participants’ homes, so interviewers could, to an extent, verify the validity of participants’ answers. Data were not available on some factors that may also be involved, such as intrafamilial violence or a lack of privacy. Also, the child‐parent relationship variables and sleep were measured at the same time as the behaviour outcome and it is possible that an element of reverse causation might explain the relationship between them and behaviour. The study did not have statistical power to analyse either changes in the level of crowding or household demographics over time. Lastly, in the SWS, the recruitment of pregnancies was necessarily over a prolonged period and the study was unable to account for potential temporal changes in housing and socio‐economic conditions between 2001 and 2010.

Our approach to causal inference using the DAG led to two different minimal sufficient adjustments sets, and we have shown analyses using both sets. Housing tenure and crowding are strongly linked and adjustment for housing tenure attenuated but did not completely remove the relationship between crowding and behavioural problems, whereas in the model adjusting for neighbourhood quality, the relationship was stronger. It is thus possible to argue that the problem lies with housing tenure rather than crowding, but we believe that our various analyses indicate that an association between crowding and behavioural problems is apparent.

### 4.4 Interpretation

The National Institute for Health and Care Excellence (NICE) recommends that vulnerable children under 5 years at risk of developing behavioural problems are identified as early as possible so that increased visits and free childcare services can be provided.^33^ This study provides support for categorising children in crowded households as “at risk” and taking action, such as referring those families to existing local support services. As maternal stress, less sleep, and strained parent‐child interactions all in part mediated the positive association between crowding and behavioural problems, intervening to influence any one of them may reduce the impact of crowding on behavioural problems. In fact, Bywater et al^34^ have already demonstrated that parenting interventions which improve parent‐child relationships can reduce behavioural problems.

In the UK, the statutory definition of overcrowding has not been updated since 1935 and it sanctions extremely overcrowded conditions.^7^, ^9^, ^35^ Problems with the statutory definition include the following: children under 1 year are not counted; people of the same gender are not entitled to their own room; living rooms and large kitchens are counted as acceptable places to sleep; and it looks at how sleeping arrangements within the premises could be organised, rather than how they are actually organised (see Figure 1 for definition).^9^, ^18^ The UK is also one of the few European nations to have no nationally agreed minimum space standards for housing.^7^ Although the effect of crowding on child behaviour is relatively modest, it does provide some support for creating space standards.^35^

Children in social housing tended to have the highest levels of crowding, so improvements in such housing to reduce crowding should be encouraged. Evaluating housing interventions that are already in place would offer tremendous research opportunities. For example, a large‐scale longitudinal study that compared two groups of households—one group where overcrowding had been alleviated compared with a group where overcrowding remained and which took into account confounding variables—would enable analysis of how crowding improvements can change behavioural trajectories.

## 5 CONCLUSIONS

Living in a more crowded home was associated with a greater risk of behavioural problems, independent of confounding factors (gender, age, single‐parent family, maternal education, receipt of benefits, social class and neighbourhood quality). The relationship was mediated in‐part by maternal stress, less sleep, and strained parent‐child interactions. Therefore, families living in crowded circumstances might benefit from greater support, or intervening on any one of the mediators may reduce the impact of crowding on behavioural problems. Crowding occurs more commonly in social housing, so increasing space in social housing would ideally be a long‐term aim.

## CONFLICT OF INTEREST

Janis Baird received research funding from Nutricia Early Life Nutrition for a specific research study which aims to improve the nutrition and vitamin D status of pregnant women and is collaborating with Iceland Foods Ltd to evaluate the impact of fruit and vegetable availability on diet. Keith Godfrey has received reimbursement for speaking at conferences sponsored by companies selling nutritional products and is part of academic research programmes that have received research funding from Abbott Nutrition, Nestec, and Danone.
