# Supplementary material for: The association between crowding within households and behavioural problems in children: Longitudinal data from the Southampton Women’s Survey
Source: Paediatr Perinat Epidemiol. 2019 Apr 29;33(3):195–203. doi: 10.1111/ppe.12550 (PMC6563047; doi:10.1111/ppe.12550)
Supplement: Supplementary file 1 [file PPE-33-195-s001.docx]

**Supplementary File eFigure 1| Box Plot Showing the Association Between Crowding in the Household and Housing Tenure**

The box represents the IQR and the whiskers represent the most extreme value within 1.5 IQR of the near quartile.
