# Supplementary material for: The association between crowding within households and behavioural problems in children: Longitudinal data from the Southampton Women’s Survey
Source: Paediatr Perinat Epidemiol. 2019 Apr 29;33(3):195–203. doi: 10.1111/ppe.12550 (PMC6563047; doi:10.1111/ppe.12550)
Supplement: Supplementary file 2 [file PPE-33-195-s002.docx]

**Supplementary File eTable 1| Multiple Imputation of Missing Values – Additional Details of Methods**

The variables included in the imputation model were either covariates or mediators included in prediction models, as well as our outcome. The variables included and information about how they were included in multiple imputation models are shown in the table below:

| **Variable** | **Type of variable** | **Transformation/Model used to predict missing data in the variable** | **How variable was entered in models to predict missing data in other variables** |
| --- | --- | --- | --- |
| Total Strengths & Difficulties score | Continuous | Square root transformation,  Linear regression | Continuous |
| Crowding in household (PPR) | Continuous | Log transformation, Log-linear regression | Continuous |
| Gender of the child | Binary | Logistic regression | Binary |
| Age of the child | Continuous | Shifted log-normal transformation,  Linear regression | Continuous |
| Currently live with partner | Binary | Logistic regression | Binary |
| Mother’s qualification level | Ordered categorical (6 categories) | Ordinal logistic Regression | 5 indicator variables |
| Receiving benefits | Binary | Logistic regression | Binary |
| Dominant household social class | Ordered categorical (6 categories) | Ordinal logistic regression | 5 indicator variables |
| Mother’s stress | Multivariable categorical  (3 categories) | Ordinal logistic regression | 2 indicator variables |
| Time sleeps for per night (hours) | Continuous | Linear regression | Continuous |
| Conflict score | Continuous | Log transformation, Predictive mean matching (k-nearest neighbours=100) | Continuous |
| Closeness score | Continuous | Shifted log-normal, Predictive mean matching (k-nearest neighbours=100) | Continuous |
| Housing Tenure | Categorical  (4 categories) | Multinomial logistic regression | 3 indicator variables |
| Neighbourhood quality | Continuous | Log transformation, Predictive mean matching (k-nearest neighbours=100) | Continuous |
