# Supplementary material for: The association between crowding within households and behavioural problems in children: Longitudinal data from the Southampton Women’s Survey
Source: Paediatr Perinat Epidemiol. 2019 Apr 29;33(3):195–203. doi: 10.1111/ppe.12550 (PMC6563047; doi:10.1111/ppe.12550)
Supplement: Supplementary file 3 [file PPE-33-195-s003.docx]

**Supplementary File eTable 2|** **Subscale Results Including Housing Tenure or Neighbourhood Quality**

| **Variable** | ***B* (95% CI) for crowding adjusted as in model 2^a^** | ***B* (95% CI) for crowding adjusted as in model 3^a^** |
| --- | --- | --- |
| Hyperactivity | 0.02 (-0.05, 0.08) | 0.04 ( -0.02, 0.10) |
| Emotional symptoms | 0.03 (-0.01, 0.07) | 0.04 (-0.0007, 0.07) |
| Conduct problems | 0.06 (0.003, 0.11) | 0.07 (0.02, 0.12) |
| Peer problems | 0.03 (-0.02, 0.07) | 0.05 (0.01, 0.09) |

**^a^** Adjustments for model 2 were: gender, age, maternal education, single parent, receipt of benefits, parental social class, and housing tenure, and for model 3 were as in model 2 but with neighbourhood quality replacing housing tenure
