# Supplementary material for: The association between crowding within households and behavioural problems in children: Longitudinal data from the Southampton Women’s Survey
Source: Paediatr Perinat Epidemiol. 2019 Apr 29;33(3):195–203. doi: 10.1111/ppe.12550 (PMC6563047; doi:10.1111/ppe.12550)
Supplement: Supplementary file 4 [file PPE-33-195-s004.docx]

**Supplementary File eTable 3| Multiple Regression Models Assessing the Relationship Between Crowding in the Household and Behavioural Problems in Children, in the Multiple Imputed Dataset (N=3,158)**

|  | **Model 1** | | | **Model 2** | | **Model 3** |
| --- | --- | --- | --- | --- | --- | --- |
| **Variable** | **B (95% CI)** | | | **B (95% CI)** | | **B (95% CI)** |
| Crowding (PPR) | 0.45 (0.33, 0.57) | | | 0.11 (-0.03, 0.24) | | 0.20 (0.07,0.32) |
| Female (vs Male) | -1.01 (-1.36, -0.67) | | | -1.05 (-1.38, -0.71) | | -1.01 (-1.35, -0.68) |
| Childs age (years) | -0.75 (-2.68, 1.19) | | | -1.50 (-3.42, 0.41) | | -1.51 (-3.41, 0.39) |
| Single parent |  | | | -0.26 (-0.92, 0.39) | | -0.60 (-1.23, 0.03) |
| Maternal education |  | | | -0.34 (-0.48, -0.19) | | -0.37 (-0.51, -0.22) |
| On benefits |  | | | 0.31 (-0.09, 0.71) | | 0.35 (-0.04, 0.75) |
| Social class (by occupation) | |  | | 0.25 (0.05, 0.45) | | 0.27 (0.07, 0.47) |
| Neighbourhood quality | |  | |  | 0.20 (0.13, 0.27) | |
| Housing Tenure  Owner occupier  Privately rented  Socially rented  Other | |  | Reference  0.07 (-0.73, 0.88)  1.56 (0.89, 2.24)  1.72 (0.68, 2.77) | |  | |
